# Interaction of AcMADS68 with transcription factors regulates anthocyanin biosynthesis in red-fleshed kiwifruit

**DOI:** 10.1093/hr/uhac252

**Published:** 2022-11-15

**Authors:** Yanfei Liu, Guowen Lv, Yaqi Yang, Kangxun Ma, Xiaolin Ren, Mingjun Li, Zhande Liu

**Affiliations:** College of Horticulture, Northwest A&F University, Yangling, 712100, Shannxi, China; College of Horticulture, Northwest A&F University, Yangling, 712100, Shannxi, China; College of Horticulture, Northwest A&F University, Yangling, 712100, Shannxi, China; College of Horticulture, Northwest A&F University, Yangling, 712100, Shannxi, China; College of Horticulture, Northwest A&F University, Yangling, 712100, Shannxi, China; College of Horticulture, Northwest A&F University, Yangling, 712100, Shannxi, China; College of Horticulture, Northwest A&F University, Yangling, 712100, Shannxi, China

## Abstract

In red-fleshed kiwifruit, anthocyanin pigmentation is a crucial commercial trait. The MYB-bHLH-WD40 (MBW) complex and other transcription factors regulate its accumulation. Herein, a new SEP gene, *AcMADS68*, was identified as a regulatory candidate for anthocyanin biosynthesis in the kiwifruit by transcriptome data and bioinformatic analyses. *AcMADS68* alone could not induce the accumulation of anthocyanin both in *Actinidia arguta* fruit and tobacco leaves. However, in combination with *AcMYBF110, AcMYB123,* and *AcbHLH1*, *AcMADS68* co-overexpression increased anthocyanin biosynthesis, whereas its silencing reduced anthocyanin accumulation. The results of the dual-luciferase reporter, firefly luciferase complementation, yeast two-hybrid and co-immunoprecipitation assays showed that AcMADS68 could interact with both AcMYBF110 and AcMYB123 but not with AcbHLH1, thereby co-regulating anthocyanin biosynthesis by promoting the activation of the target genes, including *AcANS*, *AcF3GT1*, and *AcGST1*. Moreover, *AcMADS68* also could activate the promoter of *AcbHLH1* surported by dual-luciferase reporter and yeast one-hybrid assays, thereby further amplifying the regulation signals from the MBW complex, thus resulting in enhanced anthocyanin accumulation in the kiwifruit. These findings may facilitate better elucidation of various regulatory mechanisms underlying anthocyanin accumulation and contribute to the quality enhancement of red-fleshed kiwifruit.

## Introduction

Kiwifruit (*Actinidia*, Actinidiaceae) is a popular fruit worldwide, and the red-fleshed kiwifruit is famous amongst consumers for its brilliant color and comparatively anthocyanin abundance [1]. Anthocyanins occur ubiquitously in plant tissues, wherein these are crucial for seed dispersal, pollination, protection against pathogens, and regulating responses to environmental stress [2, 3]. Moderate to high levels of anthocyanins is a crucial fruit trait for human nutrition owing to the significant antioxidant effects and potential health benefits of these molecules [4–6].

Anthocyanins synthesis has been well elucidated in several plant species. The process is catalyzed by a variety of enzymes, including cinnamate-4-hydroxylase (C4H), 4-coumaroyl:CoA-ligase (4CL), phenylalanine ammonia-lyase (PAL), chalcone flavanone isomerase (CHI), chalcone synthase (CHS), flavonoid 3′-hydroxylase (F3′H), flavanone 3-hydroxylase (F3H), leucoanthocyanidin dioxygenase (LDOX), dihydroflavonol 4-reductase (DFR), UDP-glucose:flavonoid 3-*O*-glucosyltransferase (UFGT), and glutathione S-transferase (GST) [7, 8]. The MYB-bHLH-WD40 (MBW) complex, comprising WD40, R2R3 MYB proteins, and basic helix–loop–helix (bHLH) can potentially regulate the transcription by binding to and activating the promoters of several structural genes [7–15]. Moreover, the regulatory mechanisms underlying the MBW complex’s function differ depending on the species. In *Arabidopsis*, AtTT8, AtTTG1, and AtPAP1/AtTT2 interact to form the MBW transcriptional complex, thereby stimulating the expression of late anthocyanin biosynthesis genes [7–9]. Similar interactions have been elucidated in strawberry [10] and bayberry [11]. Although bHLH proteins interact with WD40 and MYB proteins in most other plants, the MYB protein shows no interactions with WD40 in apple [12], tea [13], cotton [14], and petunia [15].

Apart from the MBW complex, other transcription factors (TFs), including ethylene-responsive factors (ERFs), WRKY, bZIP, NACs, and BBX, participate in the regulation of biosynthesis of anthocyanins through direct or indirect interactions with the MBW complex [16–22]. Recently, several reports suggest that MADS TFs are associated with anthocyanin biosynthesis regulation. For instance, *IbMADS10* participates in the developmental regulation, whereby anthocyanin accumulates in sweet potatoes, evidenced by the high correlation of its expression with those of the anthocyanin-related genes; its ectopic expression results in pigmented phenotypes in the calli [23]. *VmTDR4* and *PyMADS18* correlate with anthocyanin accumulation in bilberry [24] and European pear [25], respectively. Wang *et al.* suggest that, in pear, in response to light or temperature stress [26], *PbrMADS11* and *PbrMADS12* are involved in anthocyanin accumulation. However, convincing evidence supporting the regulation and mechanistic model of MADS TFs in the anthocyanin biosynthesis is still lacking.

In kiwifruit, structural genes of anthocyanin biosynthesis have been well studied [27–31]. Several R2R3MYB TFs, including *AcMYB5–1*, *AcMYBA1–1*, *AcMYB1*, and *AcMYB75*, are correlated with anthocyanin accumulation in the fruit [32, 33]. Wang *et al.* demonstrate that *AcbHLH42* and *AcMYB123*, which are homologs of *AtTT8* and *AtTT2*, respectively, participate in regulating anthocyanin biosynthesis by altering the expression levels of *AcF3GT1* as well as *AcANS* [34]. *AcMYBF110*, a homolog of *AcMYB110* that is specifically implicated in the red petals of kiwifruit, exhibits characteristic expression in fruit and can autonomously induce accumulation of anthocyanin in tobacco leaves by up-regulating the expressions of *DFR*, *ANS*, and *UFGT* [1, 35]. Recently, four new regulators – *AcbHLH1*, *AcbHLH5*, *AcbHLH4* and *AcWDR1 –* have been identified in kiwifruit. These four regulators interact with AcMYBF110, resulting in the formation of three MBW complexes, thereby directly or indirectly regulating anthocyanin biosynthesis [36]. Wang *et al.* suggest that differential expression and subsequent repression of MYB110 and MYB10 by SPL/ARF/SCL are responsible for differential anthocyanin accumulation in kiwifruit species [37]. However, other upstream regulatory genes involved in the accumulation of anthocyanin in the kiwifruit remain largely unknown.

In this study, transcriptome data from four inner pericarp stages and expression profiles during different developmental stages were analysed. The expression of the SEP MADS gene, *AcMADS68* (GenBank accession number ON175934) correlated remarkably with anthocyanin accumulation in the kiwifruit. Its function was confirmed by the transient transformation in *Actinidia arguta* fruit and tobacco leaves. Firefly luciferase complementation, yeast two-hybrid (Y2H) and co-immunoprecipitation (Co-IP) assays validated the interaction of AcMADS68 with AcMYBF110 or AcMYB123. Finally, the activation of anthocyanin-related gene transcription by AcMADS68 and its partners was evaluated by a dual-luciferase reporter assay and Y1H. Our findings reveal a potential regulatory mechanism via which *AcMADS68* influences kiwifruit anthocyanin biosynthesis, and may have implications for the regulatory network that underlies anthocyanin biosynthesis in other plants.

## Results

### Color and anthocyanin content in developing red-fleshed kiwifruit

**Figure 1:**
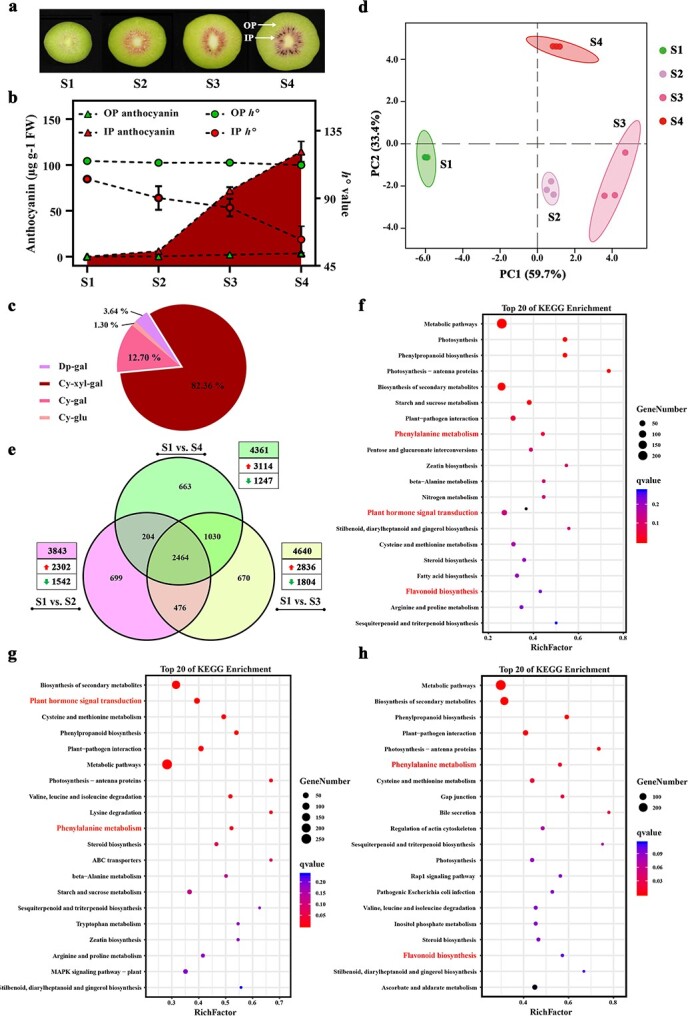
Changes in anthocyanin and RNA-seq analysis during fruit development in *Actinidia chinensis* Hongyang. **a** Fruit phenotypes at four typical stages of development. **b**–**c** Changes in the anthocyanin content and *h*-value. Dp-gal, delphinidin 3-O-galactoside; Cy-xyl-gal, cyanidin 3-*O*-xyl-galactoside; Cya-gal, cyanidin 3-*O*-galactoside; Cya-glu, cyaniding 3-*O*-glucoside. **d** Principal component analysis for the RNA-seq data. **e** Venn diagram for DEGs obtained after different comparisons. **f**–**h** Top 20 significantly enriched KEGG pathways in ‘S1 vs. S2’ (**f**), ‘S1 vs. S3’ (**g**), and ‘S1 vs. S4’ (**h**). The genes that were up-regulated are shown by the red arrows, while those that were down-regulated are denoted by the green arrows in the (e). IP, inner pericarp; OP, outer pericarp. Stages S1–S4 represent 65, 90, 115, and 140 DAP.

The flesh color of both outer and inner pericarp was green at the first fruit developmental stage [S1, 65 days after pollination (DAP), Fig. 1a] of *Actinidia chinensis* ‘Hongyang’. The flesh color of the inner pericarp changed to red in S2 (90 DAP), whereas that of the outer pericarp remained green until maturity to harvest (S4, 140 DAP). Hue angle (90 < *h*° < 180, green or yellow; 0 < *h°* < 90, red) decreased dramatically in the inner pericarp during development but the outer pericarp showed lesser changes (Fig. 1b). Cyanidin 3-*O*-xylogalactoside (Cy-xyl-gal) [1] is a major anthocyanin molecule, accounting for 82.36% of the total anthocyanins (Fig. 1c). As expected, no or only trace levels of anthocyanins were detected in S1, both in the outer and inner pericarp (Fig. 1b). Anthocyanins began to accumulate rapidly from S2 until S4 in the inner pericarp but the levels in the outer pericarp altered negligibly and were substantially lower relative to the former.

### Transcriptome analysis for fruit developmental stages

The regulatory network for anthocyanin accumulation during kiwifruit development, that is, for the four inner pericarp stages (S1 to S4) was obtained from an RNA-seq study. The numbers of clean reads (Q30 ≧ 88.58%) in all the 12 samples ranged from 19 262 389 to 28 959 486, and 91.02%–96.00% of these reads could be mapped to the kiwifruit reference genome [38, 39] (Table S1, see online supplementary material). Transcriptome characteristics of biological replicates from the same stage (Fig. S1, Table S2, see online supplementary material) were highly correlated (*r*^2^ = 0.908–0.999), indicating the reproducibility of transcriptome data and that these could be used for further analysis. Results from the principal component analysis showed that the first two principal components could explain 59.7% and 33.4% of the variance among the samples, respectively (Fig. 1d). Green (S1) and red samples (S2–S4) were separated by PC2.

### Comparison of differentially expressed genes (DEGs) across developmental stages

Comparisons were made between S1 and S2 to S4 to identify the DEGs upon anthocyanin accumulation in the kiwifruit (Fig. 1e). A total of 3843, 4640, and 4361 DEGs were identified in the three corresponding comparisons, ‘S1 vs. S2’, ‘S1 vs. S3’, and ‘S1 vs. S4’. A total of 2464 DEGs were commonly shared among the three comparisons, indicating a continued function of DEGs during all developmental stages. Upregulated DEGs were significantly higher relative to the down-regulated DEGs across comparisons (Figs 1e and S2, see online supplementary material). KEGG analysis indicated significant enrichment of the DEGs in ‘plant hormone signal transduction’, ‘phenylalanine metabolism’, and ‘flavonoid biosynthesis’ (Fig. 1f–h). These biological processes are closely associated with flavonoid biosynthesis [40]. Our results provide insight into the biological functions of the identified DEGs.

### Identification of putative TFs involved in the accumulation of anthocyanins

**Figure 2:**
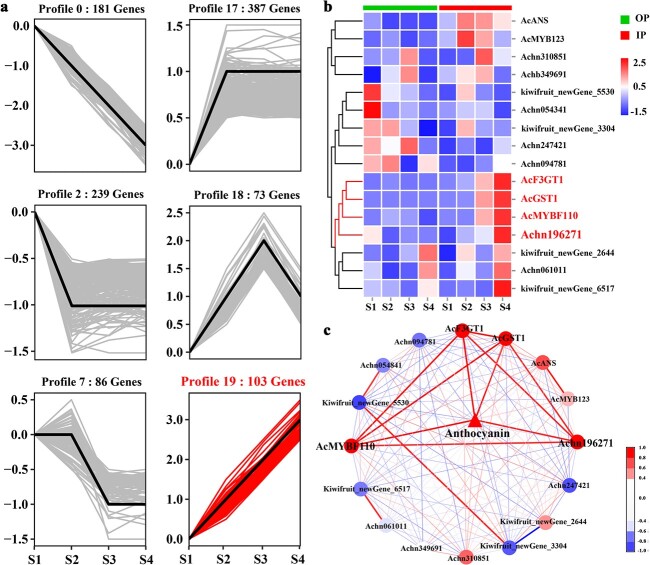
Screening of the candidate genes. **a** The profiles with *P* < 0.05 with significant genes and (**b**–**c**) expression analysis for the candidate genes. IP, inner pericarp; OP, outer pericarp. Stages S1–S4 represent 65, 90, 115, and 140 DAP.

A trend analysis was performed using 1407 DEGs (FPKM >0.5, listed in Table S3, see online supplementary material) obtained from the three comparisons to identify the putative TFs that could regulate anthocyanin accumulation during kiwifruit development. Interestingly, profile 19 (103 genes) showed changes in expression that were most similar to those observed in anthocyanin accumulation of the developing inner pericarp (Figs 1b and 2a; Fig. S3, see online supplementary material). Twelve of the genes in profile 19 were identified as TFs, and these included MYB, NAC, and MADS TFs (Table S4 and Fig. S4, see online supplementary material). Among them, *AcMYBF110* (kiwifruit_newgene_50) plays a crucial role in the accumulation of anthocyanins in kiwifruit [1, 36]. Results of qPCR indicated that Achn196271 expression correlated remarkably with those of anthocyanin-related genes, *AcMYBF110*, *AcF3GT1* [30], and *AcGST1* [29], in the inner and outer pericarp during fruit development; the highest correlation was observed with anthocyanin accumulation (*r* = 0.83, *P* < 0.01, Fig. 2b and c; Table S5, see online supplementary material). In contrast, lower correlations were observed for other candidate TFs (|r| = 0.01 to 0.58). These findings suggested that *Achn196271* may serve as an important TF involved in the accumulation of anthocyanins in kiwifruit.

### Isolation and expression of *AcMADS68*

**Figure 3:**
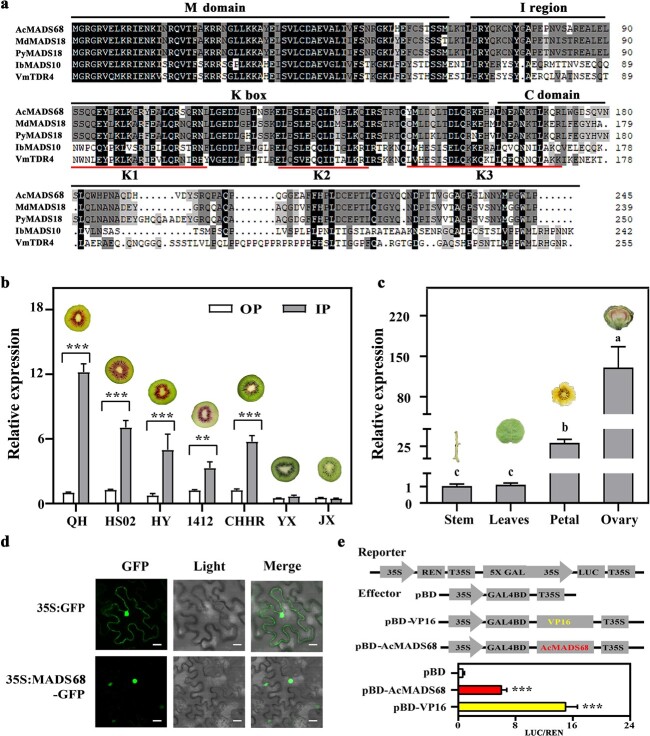
Analysis of AcMADS68. **a** Alignment of the AcMADS68 amino acid sequences and other anthocyanin-related MADS TFs found in plants. The four distinct conserved domains are denoted by the horizontal black lines. The K boxes are denoted by the horizontal red lines. The GenBank accession numbers are: AcMADS68 (ON175934), PyMADS18 (XP_009372256.1), MdMADS18 (NP_001280756.1), VmTDR4 (ACR19996.1), and IbMADS10 (ABD66305.1). Expression profiles for *AcMADS68* (**b**) in fruits from different cultivars and (**c**) various tissues of ‘Hongyang’. QH, HS02, HY, 1412, CHHR, YX, and JX represent *Actinidia chinensis* ‘Qihong’, ‘Hongshi-2’, ‘Hongyang’, and ‘150 402’, *Actinidia deliciosa* ‘ChiheRed’, ‘Yuxiang’ and ‘Jinxiang’, respectively. **d** AcMADS68 was shown to have subcellular localization in the leaves of *Nicotiana benthamiana*. Scale bar: 25 μm. **e** Transcriptional activity of *AcMADS68*.

In total, 69 MADS TFs were obtained in the *A. chinensis* Hongyang and Red5 genome database [38, 39]. *Achn196271* (*Acc32725*) was named *AcMADS68* based on its location on the chromosome (Table S6, see online supplementary material). The full-length *AcMADS68* sequence was successfully isolated and cloned from the ‘Hongyang’ fruit. This encoded a protein of 245 amino acids (Fig. 3a). Phylogenetic analysis showed that *AcMADS68* was grouped in the same clade as *MdMADS18* and *PyMADS18* (Fig. S5, see online supplementary material), two potential regulators involved in anthocyanin biosynthesis [25, 41]. Sequence alignment confirmed the presence of the four typical plant-MADS regions – the C-terminal domain, K box, I region, and M domain – in AcMADS68 and other plant anthocyanin-related MADS TFs (Fig. 3a) [42]. AcMADS68 shared a higher amino acid sequence identity with MdMADS18 (81.63%) and PyMADS18 (80.08%) but its identity was lower with IbMADS10 and VmTDR4 (44.44% and 40.08, respectively).

The transcript levels were measured in the fruits of several red-fleshed kiwifruit cultivars and across tissue types of ‘Hongyang’, to obtain the spatial and temporal expression profiles of *AcMADS68*. In the five red-fleshed kiwifruits, transcript abundance of *AcMADS68* was elevated in the inner pericarp relative to the outer pericarp, consistent with the distribution of anthocyanins (Fig. 3b); while in two green cultivars, lower transcript levels were found in bothe outer and inner pericarp. *AcMADS68* transcript levels and anthocyanin content were remarkably higher in the ovary relative to petals, stems, and leaves, wherein anthocyanin was negligibly present (Fig. 3c). Given the above data, *AcMADS68* expression correlated substantially with kiwifruit coloration and anthocyanin accumulation.

### Subcellular localization and transcriptional activity of AcMADS68

The 35S:AcMADS68-GFP fusion protein was transiently expressed in six-week-old *Nicotiana benthamiana* leaves to determine the subcellular localization of AcMADS68. The 35S:GFP protein localized to both the cytoplasm and nucleus, whereas the 35S:AcMADS68-GFP protein was exclusively detected in the nucleus, suggesting that AcMADS68 was a nuclear protein as shown in Fig. 3d.

Reporter vectors comprising 5× GAL4 activation domains upstream of the LUC gene and the effector vector (pBD, pBD-AcMADS68, and pBD-VP16) were transiently co-expressed in *N. benthamiana* leaves [43] to assess AcMADS68’s transcriptional activity *in vivo*. As with the positive control, pBD-VP16, the LUC/REN ratio increased significantly upon reporter vector’s co-expression with pBD-AcMADS68 as compared to the negative control pBD (Fig. 3e). These data suggested that *AcMADS68* may function as a transcriptional activator.

### Heterologous overexpression of *AcMADS68* and related TFs induces anthocyanin biosynthesis in tobacco leaves

**Figure 4:**
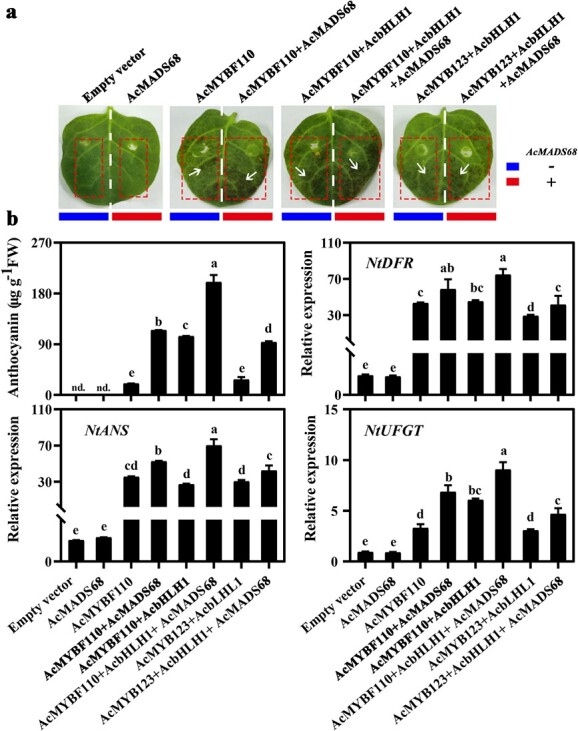
Heterologous overexpression of *AcMADS68* and related TFs induce anthocyanin biosynthesis in tobacco leaves. (**a**) Phenotype and (**b**) total anthocyanin content, along with anthocyanin-related gene expression in tobacco leaves, seven days following infiltration. Average standard errors are shown by the error bars (*n* = 3). Variations that are significant at the P < 0.05 level are denoted by lowercase letters (one-way ANOVA).

First, *AcMADS68’s* function was tested by the transient transformation of tobacco (Fig. 4a). After seven days, no pigmentation was detected in leaves infiltrated with the empty vector, *AcMADS68*, *AcMYB123,* or *AcMYB123* + *AcbHLH1* (data not shown) alone. By contrast, obvious pigmentation was found at the infiltration sites transformed with *AcMYBF110* + *AcbHLH1*, *AcMYBF110*, or *AcMYB123* + *AcbHLH1*. Notably, stronger pigmentation was seen when *AcMADS68* was co-infiltrated with the above three constructs. The anthocyanin content and transcript abundances of anthocyanin-related genes, including *NtDFR*, *NtANS*, and *NtUFGT*, were consistent with the visual phenotypes (Fig. 4b).

### Overexpression of *AcMADS68* and related TFs cause anthocyanin biosynthesis in kiwifruit

**Figure 5:**
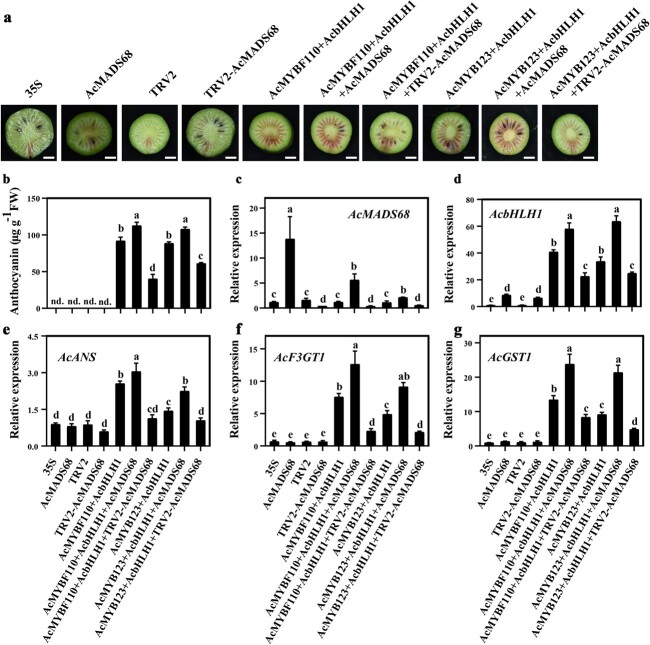
Transient overexpression or silencing of *AcMADS68* in the *A. arguta* fruit. **a** Illustration of the *A. arguta* phenotype ten days following injection. Scale bar = 0.5 cm. **b** The amount of anthocyanin found in fruit is depicted in **a**. **c**–**g** The levels of *AcMADS68* transcripts and other anthocyanin-related genes in the assessed fruit. The standard errors of the means are depicted by the error bars (*n* = 3). Variations that are significant at *P* < 0.05 are denoted by using lowercase letters (one-way ANOVA).

To further confirm the function of *AcMADS68* in anthocyanin biosynthesis in kiwifruit, the immature *A. arguta* fruit was subjected to transient transformation with *AcMADS68* and anthocyanin-related TFs [36]. No obvious pigmentation was observed after injection with the following negative controls: 35S, 35S:MADS68, TRV1 + TRV2, and TRV1 + TRV2-MADS68 (Fig. 5a). Significantly higher levels of anthocyanin accumulated in fruits co-expressing *AcMYBF110* or *AcMYB123* with *AcbHLH1* (Fig. 5a–c). Much stronger pigmentation was observed when the two combinations, *AcMYBF110* + *AcbHLH1* and *AcMYB123* + *AcbHLH1*, were co-expressed with *AcMADS68*. Reduced pigmentation was observed when these were co-expressed with the RNAi vector, TRV1 + TRV2-MADS68. The transcript levels of anthocyanin-related genes showed similar trends. Higher levels were observed when *AcMYBF110* + *AcbHLH1* or *AcMYB123* + *AcbHLH1* was co-expressed with *AcMADS68;* these were lower when *AcMYBF110* + *AcbHLH1* or *AcMYB123* + *AcbHLH1* was co-expressed with TRV1 + TRV2-MADS68 (Fig. 5e–g). Moreover, the expression of *AcbHLH1* was up-regulated upon *AcMADS68* overexpression (Fig. 5d), while those of *AcMYBF110* and *AcMYB123* remained unchanged (Fig. S6, see online supplementary material). These findings indicated that *AcMADS68* participated in the regulation of anthocyanin biosynthesis in kiwifruit.

### Interaction of AcMADS68 with other anthocyanin-related TFs

**Figure 6:**
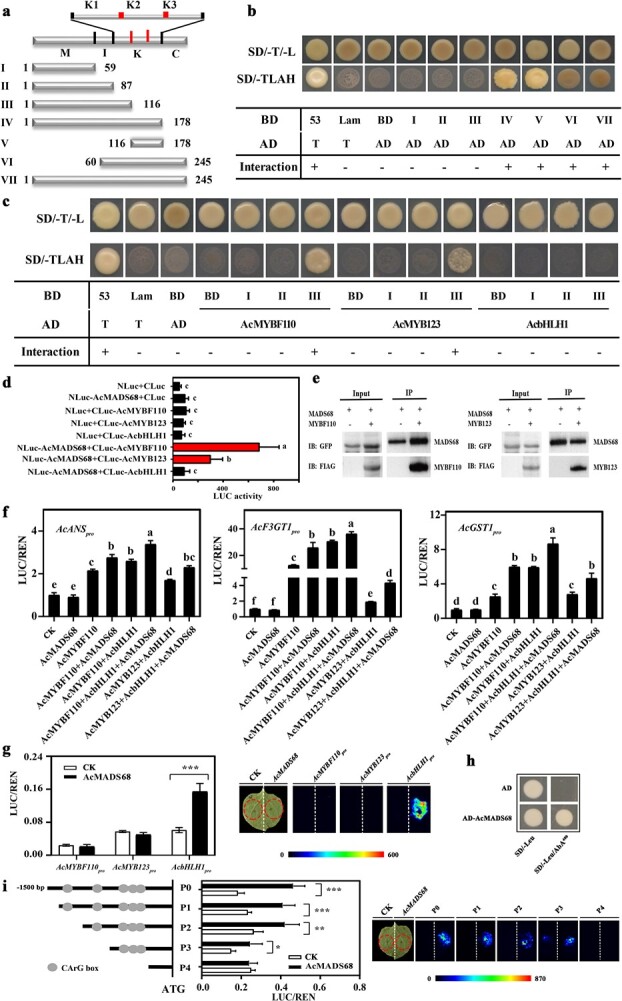
Interaction of AcMADS68 with other anthocyanin-related TFs. **a** I–VII represents distinct amino acid residues of AcMADS68. **b** Self-activation of AcMADS68 transcriptional activity. **c** Yeast-two-hybrid assay. AD, activation domain; BD, DNA-binding domain; SD/−TL, SD/Trp-Leu medium; SD/-TLAH, SD/−Trp-Leu-Ade-His medium. **d** Firefly luciferase complementation assay in *N. benthamiana* leaves. Activation of anthocyanin-related gene promoters by *AcMADS68*. **e** Co-IP assays showing that AcMYBF110-FLAG and AcMYB123-FLAG, respectively, co-immunoprecipitate with AcMADS68-GFP in *N. benthamiana* leaf. IB, immunoblotting. **f** Different combinations of promoter activation of *AcANS*, *AcF3GT1*, and *AcGST1*. **g** Activation of *AcMYBF110*, *AcMYB123*, and *AcbHLH1* promoters by *AcMADS68*. **h** AcMADS68 binding to the AcbHLH1 promoter was shown using a Y1H assay. The prey vector AD-AcMADS68 contained AcMADS68, whereas the AD-empty vector was employed as a negative control. SD/−Leu, SD medium without Leu; SD/−Leu/AbA^400^, SD medium without Leu augmented with 400 ng mL^−1^ AbA. **i***AcMADS68* stimulates the activity of five distinct *AcbHLH1* promoter segments. Mean standard errors are represented by the error bars (*n* = 3). Variations that reach the significance threshold of* P* < 0.05 are denoted by the use of lowercase letters (one-way ANOVA). ^*^P < 0.05, ^**^P < 0.01, ^***^P < 0.001 (Student’s *t*-test).

Full-length *AcMADS68* cDNA or *AcMADS68* with different N- or C-terminal deletions was cloned into pGBKT7 (Fig. 6a) to test its interactions with AcMYBF110, AcMYB123, or AcbHLH1, inserted into pGADT7. The complete amino acid sequence (VII, 1–245), N-terminal fragment IV (1–178, M domain + I region + K box), fragment V (116–178, K2-K3 box), and the C-terminal fragment (VI, 60–245) showed strong transcriptional activity in yeast (Fig. 6b). However, no transcriptional activity was found for the three N-terminal fragments, namely fragments I (the conserved M domain, residues 1–59), II (M domain + I region, 1–87), and III (M domain + I region + K1 box, 1–116). These results suggested that AcMADS68 contains a large activation domain in the K2-K3 region of its typical K-box [44, 45]. To avoid false-positive results, MADS68^1–59^ (I), MADS68^1–87^ (II), and MADS68^1–116^ (III) with no transcriptional activity were selected for Y2H analysis. When fragment I or II of AcMADS68 was co-transformed with AcMYBF110 or AcMYB123, growth was observed on SD/−T/−L medium but not on SD/−T/−L/−A/–H medium (Fig. 6c). Interestingly, when fragment III (MADS68^1–116^) was co-transformed with AcMYBF110 or AcMYB123, the yeast grew on both the SD/−T/−L medium and the SD/−T/−L/−A/–HA medium. Thus, the site of AcMADS68 interaction with AcMYBF110 or AcMYB123 was within the amino acid sequence of the K1 region (87–116). When AcbHLH1 was co-transformed with fragments I, II, or III of AcMADS68, no yeast growth was observed on the SD/−T/−L/−A/–HA medium (Fig. 6c), indicating that AcMADS68 could not interact with AcbHLH1. Collectively, these findings suggested that, in yeast, AcMADS68 interacted with the two MYB TFs (AcMYBF110 and AcMYB123) but not with AcbHLH1.

Tobacco-based firefly luciferase complementation assays were performed to confirm the results recorded from Y2H assays (Fig. 6d). Relative to the five negative controls, luciferase activity increased when NLuc-MADS68 was co-expressed with AcMYBF110-Cluc or AcMYB123-Cluc, and the combination of Nluc-MADS68 with AcMYBF110-Cluc showed the highest activity (Fig. 6d). Co-transformation of Nluc-MADS68 with AcbHLH1-Cluc resulted in lower luciferase levels, which did not differ significantly from those of the controls. These results were in line with the findings from the Y2H assays.

An *in vivo* co-immunoprecipitation (Co-IP) assay also confirmed that AcMYBF110 and AcMYB123 could be coimmunoprecipitated by AcMADS68 in total *N. benthamiana* leaf extracts (Fig. 6e).

Overall, these findings suggested that transcriptional regulatory complexes comprising MADS68-MYBF110-bHLH1 and MADS68-MYB123-bHLH1 could regulate anthocyanin biosynthesis in kiwifruit.

### Activation of anthocyanin-related gene promoters by MADS68-MYBF110-bHLH1 and MADS68-MYB123-bHLH1 complexes

To assess the interaction of AcMADS68 with the promoters of *AcANS*, *AcF3GT1*, and *AcGST1*, a dual luciferase assay was performed in tobacco leaves (Fig. 6f). None promoter could be activated when *AcMADS68* was expressed alone relative to the empty vector (SK). All three promoters were clearly activated upon *AcMYBF110* expression alone and upon co-expression of *AcMYBF110* + *AcbHLH1* or *AcMYB123* + *AcbHLH1*. Interestingly, the activation effects were enhanced by co-expression with *AcMADS68*. These findings indicated that the MADS68-MYBF110-bHLH1 or MADS68-MYB123-bHLH1 complex could directly activate the promoters of *AcANS*, *AcF3GT1*, and *AcGST1*, resulting in the accumulation of anthocyanins in the kiwifruit.

A dual-luciferase and Y1H assay demonstrated that the promoter of *AcbHLH1I*, but not *AcMYBF110* or *AcMYB123*, was activated directly by *AcMADS68* (Fig. 6g–h). To unravel the roles of different CArG motifs in the *AcbHLH1* promoter, five different fragments (P0 to P4) were constructed to perform dual-luciferase assays (Fig. 6i). For the promoter fragment P4 that lacked the CArG motif, no obvious activation was observed. All of the other promoter fragments (P0–P3), comprising different numbers of CArG motifs showed activation to various extents.

## Discussion

### Transcriptomics shows that *AcMADS68* is crucial to the regulation of anthocyanin biosynthesis in kiwifruit

The red-fleshed or red peel trait owing to high anthocyanin abundance is a crucial quality index. Several TFs participate in anthocyanin biosynthesis in the fruit. In apples, *MYB10* and *MYB110a* are responsible for the two distinct phenotypes of red-flesh apples (Types I and II) [46–49]. Studies have characterized additional TFs (*MdHY5*, *MdERF38*, *MdEIL1*, *MdERF17*, and *MdPIF7*) that regulate anthocyanin accumulation in apple [19, 21, 50–53]. Two long noncoding RNAs, *MdLNC499* and *MdLNC610*, can induce anthocyanin accumulation in apples under high light [54, 55]. In blood-fleshed peach, the NAC TF, *BL,* regulates anthocyanin biosynthesis by forming a heterodimer with *PpNAC1*, thereby activating the *PpMYB10.1* promoter [17]. In pear, *PyERF3* and *PyWRKY26* interact with *PybHLH3, PyMYB114*, and *PyMYB10*, thus co-regulating anthocyanin biosynthesis [18, 56]. The B-box protein, BBX16, positively regulates anthocyanin biosynthesis under high light by activating *PyMYB10* in red pear [20]. In strawberries, *FaMYB10* plays a vital role in anthocyanin biosynthesis [57, 58], and *FaRAV1* promotes anthocyanin accumulation by directly activating the promoters of *FaMYB10* and genes involved in the anthocyanin pathway [16].

In kiwifruit, several identified TFs are known to function as regulators of anthocyanin biosynthesis. *AcMYBA1–1* and *AcMYB5–1* enhance anthocyanin accumulation at low storage temperatures by upregulating the expression of genes in the anthocyanin pathway [32]. In *Arabidopsis*, overexpression of *AcMYB75*, whose expression during the fruit development of ‘Hongyang’, correlates remarkably with anthocyanin accumulation, can enhance anthocyanin accumulation [33]. AcMYB123, interacting with AcbHLH42, regulates anthocyanin accumulation by activating the promoter of *AcF3GT1* in kiwifruit [34]. Herein, *AcMYBF110* was found to play a crucial regulatory role in anthocyanin accumulation via the activation of the promoters of several genes in the anthocyanin pathway, especially *AcANS*, *AcF3GT1*, and *AcGST1*. AcMYBF110 interacts with both AcWDR1 and AcbHLH1/4/5, thereby forming three distinct MBW complexes that hierarchically regulate anthocyanin biosynthesis in the red-fleshed kiwifruit [36]. Overall, these reports focus on the functional regulation of MYB TFs in anthocyanin biosynthesis in the kiwifruit.

Transcriptomics has been widely used for identifying potential regulators of fruit quality traits in horticultural crops [59–62]. In this study, RNA-seq data, trend analysis, and expression profiles during multiple developmental stages helped us newly identify the SEP MADS TF, *AcMADS68* (Figs 1 and 2). The expression of *AcMADS68* correlated remarkably with color and the accumulation of anthocyanins in kiwifruit (Fig. 3b and c). *AcMADS68*’s function was also confirmed by transiently transforming *A. arguta* fruit and tobacco leaves (Figs 4 and 5). Thus, we characterized the involvement of *AcMADS68* in the regulation of anthocyanin biosynthesis in kiwifruit.

### The interaction of AcMADS68 with AcMYBF110 and AcMYB123 regulates anthocyanin biosynthesis

A regulatory network incorporating TFs and the MBW complex controls anthocyanin biosynthesis [8, 9]. Several TFs can regulate anthocyanin biosynthesis by promoting the activation of positive MYB or MBW complexes. For example, *FaRAV1* enhances the accumulation of anthocyanin by the activation of genes in the anthocyanin pathway and *FaMYB10* [16]. This finding is similar to those reported for peach BL [17], apple *MdHY5* and *MdHB1* [21, 22], and pear BBX16 [20]. Some anthocyanin activators, including *Arabidopsis AtTCP3* [63], apple *MdERF1B* and *MdERF38* [50], and pear *PyERF3* and *PyWRKY26* [18, 56], typically interact with anthocyanin-activating MYBs and enhance the activation of MBW complexes, thereby promoting anthocyanin accumulation. In addition, a few TFs, including *PpMYB18*, *MdMYB16/17*, and *AtMYBL2* [64–67], regulate anthocyanin accumulation by competing with MYB or bHLH, thus interrupting the MBW complex. Herein, the results of firefly luciferase complementation and Y2H assays demonstrated that AcMADS68 interacted separately with AcMYBF110 and AcMYB123 but not with AcbHLH1 (Fig. 6a–e). AcMADS68 did not activate the promoter of *AcMYBF110* or *AcMYB123* (Fig. 6h–i). This result differs from that reported previously by Jaakola *et al.*, whereby *VmTDR4* attenuated anthocyanin accumulation, either by indirect or direct control of the R2R3 MYB family during ripening of bilberry [24]. Phylogenetic analysis suggested that *AcMADS68* was a SEP MADS gene, and *VmTDR4* belonged to the AP1 subfamily (Fig. S5, see online supplementary material). They were placed in different clades and shared 40.08% identity (Fig. 3a), indicating differential regulation of anthocyanin, similar to other functions in plants [68]. This is supported by the differential regulation of anthocyanin accumulation by *FaRAV1* and *FaERF85* in strawberry [16].

The MBW complex and other anthocyanin-related TFs in several plants promote anthocyanin accumulation by stimulating gene expression in the anthocyanin pathway [9]. As shown in Figs 4 and 5, genes in the anthocyanin pathway were significantly upregulated when *AcMADS68* was co-expressed with *AcMYBF110*, *AcMYBF110* + *AcbHLH1*, or *AcMYB123* + *AcbHLH1* in *A. arguta* fruit or tobacco leaves, which was consistent with the results of anthocyanin accumulation. Dual-luciferase reporter assays showed that promoter activation of *AcANS*, *AcF3GT1*, and *AcGST1* was strongly enhanced by overexpression of the AcMADS68-AcMYBF110-AcbHLH1 or AcMADS68-AcMYB123-AcbHLH1 complex (Fig. 6f). Hierarchical and feedback mechanisms between the MBW complex was an important feature of anthocyanin regulation [8, 9, 36]. For example, overexpression of AtPAP1/AtTT2 in Arabidopsis increased expression of AtTT8 [8]. In kiwifruit, overexpression of AcbHLH1 also could induce increases in AcMYBF110 transcription [36]. In this study, *AcMADS68* activated the promoter of *AcbHLH1*, thereby regulating its expression to amplify the regulation signals of the MADS68-MYBF110-bHLH1 or MADS68-MYB123-bHLH1 (Figs 5d and 6g–i).

**Figure 7:**
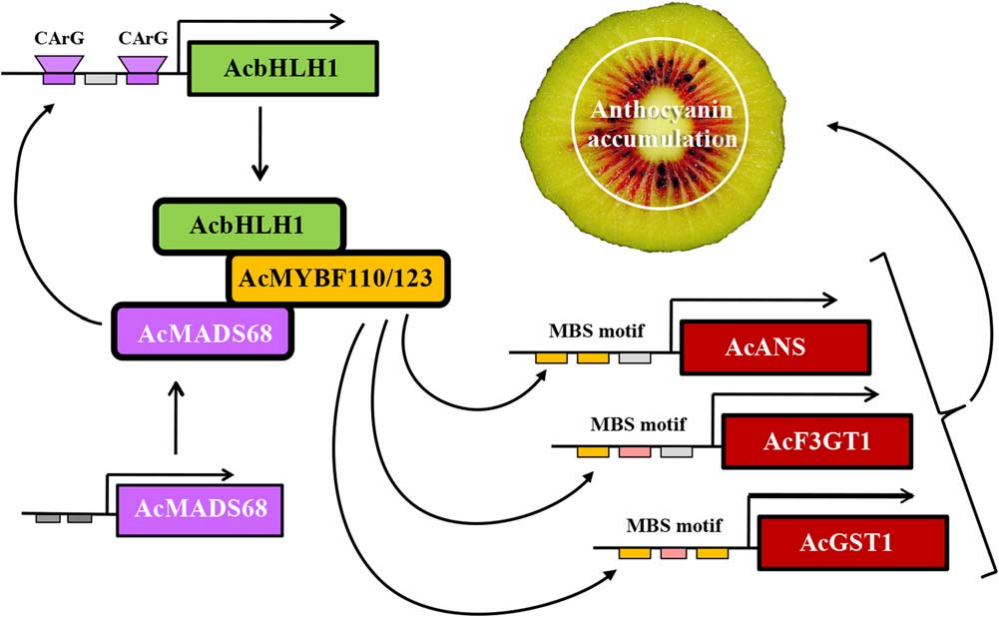
Anthocyanin biosynthesis of kiwifruit is regulated by AcMADS68 in two ways. Firstly, AcMADS68 interacted separately with AcMYBF110 and AcMYB123 resluting in stabilizing the formation of the MBW complex, thus promoting anthocyanin biosynthesis by upregulating the expression of anthocyanin related genes. On the other hand, the promoter of AcbHLH1 was directly activated by AcMADS68 and then amplified the MBW complex’s regulation signals, resulting in enhanced accumulation of anthocyanins in kiwifruit.

In this study, the TF AcMADS68, which interacted separately with AcMYBF110 and AcMYB123, stabilized the formation of the MBW complex and upregulated the expression of *AcANS*, *AcF3GT1*, and *AcGST1*, thus promoting anthocyanin biosynthesis (Fig. 7). *AcMADS68* directly activated the promoter of *AcbHLH1*, thus amplifying the MBW complex’s regulation signals and resulting in enhanced accumulation of anthocyanins in the red-fleshed kiwifruit. These findings provide novel insight into the regulatory network of anthocyanin biosynthesis and may contribute to the production of new kiwifruit cultivars with high anthocyanin abundance.

## Materials and methods

### Plant materials

Different tissues of *A. chinensis* cv. ‘Hongyang’, including stems, leaves, petals, and ovaries, were obtained at the flowering stage. The fruits of ‘Hongyang’ were sampled at four developmental stages (65, 90, 115, and 140 DAP), and mature fruits (140 DAP) were collected from four additional red-fleshed cultivars, namely *A. chinensis* ‘Qihong’, ‘Hongshi-2’, ‘150 402’, and *A. deliciosa* ‘ChiheRed’, ‘Yuxiang’, and ‘Jinxiang’. All samples were collected from the National Center for Kiwifruit Breeding in Mei County (East longitude 107°39'~108°00', North latitude 33°59'~34°19'), Shannxi Province, China. Three replicates of each tissue were frozen instantly in liquid nitrogen and preserved at −80°C for subsequent studies.

### Extraction and evaluation of anthocyanin content

Powdered samples (about 1 g) were homogenized in 5 ml 1% (v/v) HCl/methanol and incubated for 24 hours in darkness at 4°C. HPLC (1260, Agilent, Palo Alto，California，USA) coupled to an InertsilODS-3 column (5.0 μm, 250 × 4.6 mm, GL Sciences Inc., Tokyo, Japan) and a diode array detector (Agilent Technology, Palo Alto, CA, USA) was used to assess the extracts after 10 minutes of centrifugation the supernatant at 8000$\times$*g* and filtering it using a 0.22-μm filter [1]. Mobile phases were 10% formic acid and 1.36% water in acetonitrile (B) and 10% (v/v) formic acid/water (A). The gradient was 95% A (0 min), 85% A (25 min), 78% A (42 min), 64% A (60 min), and 95% A (65 min). Monitoring was at 520 nm accompanied by a post-run period of 10 minutes. Data were obtained from three replicates per sample.

### RNA-seq and data analyses

RNA library preparation and sequencing were performed as described previously [69] using inner pericarps at the four developmental stages (S1 to S4). The concentration, integrity, and purity of the resulting RNA were detected with appropriate instruments and methods. HISAT2 v2.0.5 and HTSeq v0.7.2 were used to align and count the clean reads to the kiwifruit reference genome, and the FPKM values were estimated from the read counts mapped onto the genes and the gene lengths. DESeq2 package in R (1.18.0) was used to analyse identified significant DEGs with the threshold fixed at fold change >2 and *P* < 0.05 [70, 71].

The omicshare website (www.omicshare.com/tools/Home/Soft/pathwaygseasenior) was used to conduct a statistical analysis of the enrichment of DEGs in KEGG pathways. Among them, 1407 genes (FPKM>0.5) were subjected to trend analysis (www.omicshare.com/tools/Home/Soft/trend).

### Expression analyses by qPCR

The Plant RNA Kit (Omega Biotek, Norcross, GA, USA) was employed to isolate around 1 μg of total RNA, which was then subjected to cDNA synthesis utilizing the Prime Script RT reagent kit (TaKaRa, Dalian, China). Additionally, qPCR was performed with the SYBR Premix Ex TaqII Kit (TaKaRa, Dalian, China). The conditions for amplification entailed one cycle at 95°C for 40 s followed by 40 cycles at 95°C for 30 s, and 58°C for 30 s. Primers applied in this research are detailed in Table S7 (see online supplementary material). By employing the 2^−ΔΔCt^ method, data were normalized relative to actin and PP2A [72]. We performed each analysis three times (three PCRs for each biological duplicate, and three times for each gene in the sample) with biological replicates.

### Extraction of *AcMADS68* and sequence analyses

The whole set of 69 MADS TF sequences was derived from the kiwifruit genome database (http://kiwifruitgenome.org/home). After matching the protein sequences with ClustalW and making any required adjustments manually, a phylogenetic tree was generated in MEGA 5.0 utilizing the neighbor-joining technique and 1000 bootstrap iterations [73].

### *AcMADS68* subcellular localization

The coding region of *AcMADS68* that did not include the stop codon was subjected to PCR amplification, after which it was inserted into the *pCAMBIA2300* vector in frame with the GFP sequence (Table S7 contains a listing of the primer sequences, see online supplementary material). The fused constructs, 35S:AcMADS68-GFP and 35S:GFP, were transmuted into *A. tumefaciens* strain, GV3101. *A. tumefaciens* strain (OD_600_ = 0.6–0.8) was individually introduced into leaves of *N. benthamiana* that are six weeks old. Using a confocal laser scanning microscope (TCS SP8 SR, Leica) the intensity of the fluorescence signal was evaluated 48 hours following a transformation.

### Transient luciferase assays

The reporter, which is a derivation of pGreenII 0800-LUC and contains 5× GAL4 AD upstream of the minimum CaMV35S promoter as well as the LUC gene, was utilized for determining the level of transcriptional activity (Fig. 3e). This effector vector, pBD-AcMADS68, was generated by cloning the *AcMADS68* coding sequence (CDS) into the pBD vector, which contains the GAL4 DNA-binding site. The negative control was the pBD (empty vector) whereas pBD-VP16 was a positive control.

The CDSs of *AcMADS68*, as well as those of other TFs, were introduced into the pGreenII 62-SK vector, resulting in the formation of the effectors [74] for DNA-promoter interaction assays (Table S7 outlines the primer sequences, see online supplementary material), and the reporters were obtained by inserting the promoters of genes associated with anthocyanin into the pGreenII 0800-LUC vector. As a negative control, we used the SK vector.

With the pSoup helper plasmid, each of the generated constructs was transferred into the GV3101 strain of *A. tumefaciens*. Injections of several *Agrobacterium* mixtures (OD600 0.8) containing the reporter and effectors were made into the leaves of *N. benthamiana* that had been growing for six weeks. Forty-eight hours following the transformation, luciferase activity was measured with the Dual-Luciferase Reporter Assay System (Promega, Madison, Wisconsin, USA) in compliance with the specifications provided by the manufacturer. To conduct a dual-luciferase experiment, the leaves that had been transformed were both sprayed and immersed in a solution consisting of 1.0 mM luciferin (Promega) in 0.01% Triton X-100. After five minutes of being in the dark, the fluorescence was quenched, and the LUC pictures were acquired utilizing a low-light CCD imaging device that was cooled (Lumazone Pylon2048B; Roper Scientific, Trenton, NJ, USA).

### Transient expression in *A. arguta* fruit and tobacco leaves

By using the pSoup helper plasmid, the pGreenII 62SK-AcMADS68, AcMYBF110, AcMYB123, and AcbHLH1, and pGreenII 62-SK empty vector were introduced into the GV3101 *A. tumefaciens* strain for transient overexpression. The TRV2-MADS68 construct was created by inserting a coding sequence from *AcMADS68* into pTRV2 to silence gene expression. Various combinations of these strains (about 400 μL) were introduced into either *N. tabacum* leaves or immature *A. arguta* fruit. The injected tobacco leaves were sampled seven days after transformation and photographed. The infiltrated *A. arguta* fruit was left on the vine for an additional ten days, imaged, and harvested for measuring anthocyanin content and extracting RNA.

### Y2H assays

The full-length CDS of *AcMADS68* and six partial fragments of *AcMADS68* (MADS68^1–59^, MADS68^1–87^, MADS68^1–116^, MADS68^1–178^, MADS68^116–87^, and MADS68^60–245^) were inserted into pDBKT7. The resulting constructs were transformed into Y2HGold with the Matchmaker Gold Yeast Two-Hybrid System (Clontech, Mountain View, CA, USA). The transformed yeast cells were cultured on SD/−Leu/−Trp/-His/−Ade and SD-Trp-Leu media to assess the transactivation of AcMADS68. The full-length CDSs of *AcMYBF110*, *AcMYB123*, and *AcbHLH1* were inserted into pGADT7 for Y2H assays. Three pGBK fusion constructs (MADS68^1–59^, MADS68^1–87^, and MADS68^1–116^) and the pGADT7 fusion construct were co-transformed into Y2HGOLD and plated on the SD-Trp-Leu medium. PCR was used to verify the viability of the positive colonies, which were then subjected to further testing on the SD-Trp-Leu and SD-Leu-Trp media. After three or four days, images are captured.

### Firefly luciferase complementation assays

The *N. benthamiana* leaves were tested using the firefly luciferase complementation assay at the six-week mark, following the protocol established earlier [75]. Full-length CDSs of *AcMADS68*, *AcMYBF110*, *AcMYB123*, and *AcbHLH1* were inserted into the binary vector pCambia1300cLUC (35S:CLuc) or pCambia1300nLUC (35S:NLuc). Preparation, as well as infiltration, of *Agrobacterium* were performed following the protocol for the transient expression assay. We used the Steady-Glo Luciferase Assay System (Promega) to measure the firefly luciferase activity of the samples.

### Co-Immunoprecipitation (Co-IP) assay

CDs of AcMYBF110 and AcMYB123 were cloned into the pCAMBIA35s-4 × Myc-MCS-3 × FLAG vector. AcMADS68-GFP was also used in the Co-IP assay. The leaves of *N. benthamiana* were harvested 48 hours after being co-infiltrated with various Agrobacterium mixtures*.* Anti-Flag antibodies (TransGen, Haidian District, Beijing, China) and anti-GFP antibodies (TransGen, Haidian District, Beijing, China) were used in Co-IP experiments.

### Yeast one-hybrid (Y1H)

We cloned the promoter of AcbHLH1 into pAbAi-baits (Clontech) and the CDS of AcMADS68 into the pGADT7 vector. After transforming the linearized pAbAi-baits into Y1H Gold, the bait strain’s minimal inhibitory concentration for Aureobasidin A (AbA) was determined by using the SD/-Ura and SD/-Ura/AbAx medium. Next, yeast cells harboring bait constructs were transfected with either the empty vector (AD) (the control) or AD-AcMADS68 and thereafter spotted on SD/−Leu medium with the least amounts of the AbA antibiotic after being diluted with a 10-fold dilution cycle.

## Acknowledgments

This work was supported by grants from the National Natural Science Foundation (Grant No. 32102312), the modern agricultural industry technology system (Grant No. CARS-26), and the National Forestry and Grassland Extension Project (Grant No. K3130219012). The authors thank Prof. Wangjin Lu for providing the pBD-related vectors.

## Author contributions

Y.L., G.L., Y.Y., and K.M. performed experiments and analyses, and Y.L. wrote and revised the manuscript. X.R. and M.L. helped revise the manuscript. Z.L. designed the experiments and revised the manuscript. All authors have participated in this research and approved the final manuscript.

## Data availability

The sequence data of AcMADS68 were submitted to the NCBI database (accession number ON175934). The other data for this article are available in the article or in its online supplementary material.

## Conflict of interest

The authors declare no potential conflicts of interest.

## Supplementary data

Supplementary data is available at *Horticulture Research* online.

## Supplementary Material

Web_Material_uhac252

## References

[1] Liu Y , Zhou B, Qi Y et al. Expression differences of pigment structural genes and transcription factors explain flesh coloration in three contrasting kiwifruit cultivars. *Front Plant Sci.* 2017;8:1507. 28919902 10.3389/fpls.2017.01507 PMC5586210

[2] Winkel-Shirley B . Flavonoid biosynthesis. A colorful model for genetics, biochemistry, cell biology, and biotechnology. *Plant Physiol.* 2001;126:485–93. 11402179 10.1104/pp.126.2.485 PMC1540115

[3] Xie XB , Li S, Zhang RF et al. The bHLH transcription factor MdbHLH3 promotes anthocyanin accumulation and fruit colouration in response to low temperature in apples. *Plant Cell Environ.* 2012;35:1884–97. 22519753 10.1111/j.1365-3040.2012.02523.x

[4] Butelli E , Titta L, Giorgio M et al. Enrichment of tomato fruit with health-promoting anthocyanins by expression of select transcription factors. *Nat Biotechnol.* 2008;26:1301–8. 18953354 10.1038/nbt.1506

[5] Liu Y , Qi Y, Chen X et al. Phenolic compounds and antioxidant activity in red- and in green-fleshed kiwifruits. *Food Res Int.* 2019;116:291–301. 30716948 10.1016/j.foodres.2018.08.038

[6] Petroni K , Pilu R, Tonelli C. Anthocyanins in corn: a wealth of genes for human health. *Planta.* 2014;240:901–11. 25106530 10.1007/s00425-014-2131-1

[7] Baudry A , Heim MA, Dubreucq B et al. TT2, TT8, and TTG1 synergistically specify the expression of BANYULS and proanthocyanidin biosynthesis in Arabidopsis thaliana. *Plant J.* 2004;39:366–80. 15255866 10.1111/j.1365-313X.2004.02138.x

[8] Xu W , Grain D, Bobet S et al. Complexity and robustness of the flavonoid transcriptional regulatory network revealed by comprehensive analyses of MYB-bHLH-WDR complexes and their targets in Arabidopsis seed. *New Phytol.* 2014;202:132–44. 24299194 10.1111/nph.12620

[9] Xu W , Dubos C, Lepiniec L. Transcriptional control of flavonoid biosynthesis by MYB-bHLH-WDR complexes. *Trends Plant Sci.* 2015;20:176–85. 25577424 10.1016/j.tplants.2014.12.001

[10] Schaart JG , Dubos C, Romero de la Fuente I et al. Identification and characterization of MYB-bHLH-WD40 regulatory complexes controlling proanthocyanidin biosynthesis in strawberry (Fragaria x ananassa) fruits. *New Phytol.* 2013;197:454–67. 23157553 10.1111/nph.12017

[11] Liu X , Feng C, Zhang M et al. The MrWD40-1 gene of Chinese bayberry (Myrica rubra) interacts with MYB and bHLH to enhance anthocyanin accumulation. *Plant Mol Biol Report.* 2013;31:1474–84.

[12] An XH , Tian Y, Chen KQ et al. The apple WD40 protein MdTTG1 interacts with bHLH but not MYB proteins to regulate anthocyanin accumulation. *J Plant Physiol.* 2012;169:710–7. 22405592 10.1016/j.jplph.2012.01.015

[13] Sun B , Zhu Z, Cao P et al. Purple foliage coloration in tea (Camellia sinensis L.) arises from activation of the R2R3-MYB transcription factor CsAN1. *Sci Rep.* 2016;6:32534. 27581206 10.1038/srep32534 PMC5007479

[14] Yan Q , Wang Y, Li Q et al. Up-regulation of GhTT2-3A in cotton fibres during secondary wall thickening results in brown fibres with improved quality. *Plant Biotechnol J.* 2018;16:1735–47. 29509985 10.1111/pbi.12910 PMC6131414

[15] Albert NW , Davies KM, Lewis DH et al. A conserved network of transcriptional activators and repressors regulates anthocyanin pigmentation in eudicots. *Plant Cell.* 2014;26:962–80. 24642943 10.1105/tpc.113.122069 PMC4001404

[16] Zhang ZY , Shi Y, Ma Y et al. The strawberry transcription factor FaRAV1 positively regulates anthocyanin accumulation by activation of FaMYB10 and anthocyanin pathway genes. *Plant Biotechnol J.* 2020;18:2267–79. 32216018 10.1111/pbi.13382 PMC7589338

[17] Zhou H , Lin-Wang K, Wang H et al. Molecular genetics of blood-fleshed peach reveals activation of anthocyanin biosynthesis by NAC transcription factors. *Plant J.* 2015;82:105–21. 25688923 10.1111/tpj.12792

[18] Yao G , Ming M, Allan AC et al. Map-based cloning of the pear gene MYB114 identifies an interaction with other transcription factors to coordinately regulate fruit anthocyanin biosynthesis. *Plant J.* 2017;92:437–51. 28845529 10.1111/tpj.13666

[19] Mao Z , Jiang H, Wang S et al. The MdHY5-MdWRKY41-MdMYB transcription factor cascade regulates the anthocyanin and proanthocyanidin biosynthesis in red-fleshed apple. *Plant Sci.* 2021;306:110848. 33775373 10.1016/j.plantsci.2021.110848

[20] Bai S , Tao R, Tang Y et al. BBX16, a B-box protein, positively regulates light-induced anthocyanin accumulation by activating MYB10 in red pear. *Plant Biotechnol J.* 2019;17:1985–97. 30963689 10.1111/pbi.13114 PMC6737026

[21] An JP , Qu FJ, Yao JF et al. The bZIP transcription factor MdHY5 regulates anthocyanin accumulation and nitrate assimilation in apple. *Hortic Res.* 2017;4:17023. 28611922 10.1038/hortres.2017.23 PMC5461414

[22] Jiang YH , Liu C, Yan D et al. MdHB1 down-regulation activates anthocyanin biosynthesis in the white-fleshed apple cultivar 'Granny Smith'. *J Exp Bot.* 2017;68:1055–69. 28338881 10.1093/jxb/erx029

[23] Lalusin AG , Nishita K, Kim SH et al. A new MADS-box gene (IbMADS10) from sweet potato (Ipomoea batatas (L.) lam) is involved in the accumulation of anthocyanin. *Mol Gen Genomics.* 2006;275:44–54. 10.1007/s00438-005-0080-x 16333667

[24] Jaakola L , Poole M, Jones MO et al. A SQUAMOSA MADS box gene involved in the regulation of anthocyanin accumulation in bilberry fruits. *Plant Physiol.* 2010;153:1619–29. 20566708 10.1104/pp.110.158279 PMC2923880

[25] Wu J , Zhao G, Yang YN et al. Identification of differentially expressed genes related to coloration in red/green mutant pear (Pyrus communis L.). *Tree Genet Genomes.* 2013;9:75–83.

[26] Wang R , Ming M, Li J et al. Genome-wide identification of the MADS-box transcription factor family in pear (Pyrus bretschneideri) reveals evolution and functional divergence. *PeerJ.* 2017;5:e3776. 28924499 10.7717/peerj.3776 PMC5598432

[27] Li YK , Fang J, Qi X et al. A key structural gene, AaLDOX, is involved in anthocyanin biosynthesis in all red-fleshed kiwifruit (Actinidia arguta) based on transcriptome analysis. *Gene.* 2018;648:31–41. 29309888 10.1016/j.gene.2018.01.022

[28] Liu Y , Liu J, Qi Y et al. Identification and characterization of AcUFGT6b, a xylosyltransferase involved in anthocyanin modification in red-fleshed kiwifruit (Actinidia chinensis). *Plant Cell, Tissue Organ Cult.* 2019;138:257–71.

[29] Liu Y , Qi Y, Zhang A et al. Molecular cloning and functional characterization of AcGST1, an anthocyanin-related glutathione S-transferase gene in kiwifruit (Actinidia chinensis). *Plant Mol Biol.* 2019;100:451–65. 31079310 10.1007/s11103-019-00870-6

[30] Liu Y , Zhou B, Qi Y et al. Biochemical and functional characterization of AcUFGT3a, a galactosyltransferase involved in anthocyanin biosynthesis in the red-fleshed kiwifruit (Actinidia chinensis). *Physiol Plant.* 2018;162:409–26. 29057484 10.1111/ppl.12655

[31] Montefiori M , Espley RV, Stevenson D et al. Identification and characterisation of F3GT1 and F3GGT1, two glycosyltransferases responsible for anthocyanin biosynthesis in red-fleshed kiwifruit (Actinidia chinensis). *Plant J.* 2011;65:106–18. 21175894 10.1111/j.1365-313X.2010.04409.x

[32] Li B , Xia Y, Wang Y et al. Characterization of genes encoding key enzymes involved in anthocyanin metabolism of kiwifruit during storage period. *Front Plant Sci.* 2017;8:341. 28344589 10.3389/fpls.2017.00341 PMC5344892

[33] Li W , Ding Z, Ruan M et al. Kiwifruit R2R3-MYB transcription factors and contribution of the novel AcMYB75 to red kiwifruit anthocyanin biosynthesis. *Sci Rep.* 2017;7:16861. 29203778 10.1038/s41598-017-16905-1 PMC5715094

[34] Wang L , Tang W, Hu Y et al. AMYB/bHLHcomplex regulates tissue–specific anthocyanin biosynthesis in the inner pericarp of red–centered kiwifruitActinidia chinensiscv. Hongyang. *Plant J.* 2019;99:359–78. 30912865 10.1111/tpj.14330

[35] Fraser LG , Seal AG, Montefiori M et al. An R2R3 MYB transcription factor determines red petal colour in an Actinidia (kiwifruit) hybrid population. *BMC Genomics.* 2013;14:28. 23324587 10.1186/1471-2164-14-28 PMC3618344

[36] Liu YF , Ma K, Qi Y et al. Transcriptional regulation of anthocyanin synthesis by MYB-bHLH-WDR complexes in kiwifruit (Actinidia chinensis). *J Agric Food Chem.* 2021;69:3677–91. 33749265 10.1021/acs.jafc.0c07037

[37] Wang WQ , Moss SMA, Zeng L et al. The red flesh of kiwifruit is differentially controlled by specific activation-repression systems. *New Phytol.* 2022;235:630–45. 35348217 10.1111/nph.18122

[38] Pilkington SM , Crowhurst R, Hilario E et al. A manually annotated Actinidia chinensis var. chinensis (kiwifruit) genome highlights the challenges associated with draft genomes and gene prediction in plants. *BMC Genomics.* 2018;19:257. 29661190 10.1186/s12864-018-4656-3 PMC5902842

[39] Yue J , Liu J, Tang W et al. Kiwifruit genome database (KGD): a comprehensive resource for kiwifruit genomics. *Hortic Res.* 2020;7:117. 32821400 10.1038/s41438-020-0338-9 PMC7395147

[40] Jaakola L . New insights into the regulation of anthocyanin biosynthesis in fruits. *Trends Plant Sci.* 2013;18:477–83. 23870661 10.1016/j.tplants.2013.06.003

[41] Tian Y , Dong Q, Ji Z et al. Genome-wide identification and analysis of the MADS-box gene family in apple. *Gene.* 2015;555:277–90. 25447908 10.1016/j.gene.2014.11.018

[42] Kaufmann K , Melzer R, Theissen G. MIKC-type MADS-domain proteins: structural modularity, protein interactions and network evolution in land plants. *Gene.* 2005;347:183–98. 15777618 10.1016/j.gene.2004.12.014

[43] Lu S , Zhang Y, Zhu K et al. The citrus transcription factor CsMADS6 modulates carotenoid metabolism by directly regulating Carotenogenic genes. *Plant Physiol.* 2018;176:2657–76. 29463773 10.1104/pp.17.01830 PMC5884614

[44] Sun CH , Yu JQ, Wen LZ et al. Chrysanthemum MADS-box transcription factor CmANR1 modulates lateral root development via homo−/heterodimerization to influence auxin accumulation in Arabidopsis. *Plant Sci.* 2018;266:27–36. 29241564 10.1016/j.plantsci.2017.09.017

[45] Davies B , EgeaCortines M, Silva ED et al. Multiple interactions amongst floral homeotic MADS box proteins. *EMBO J.* 1996;15:4330–43. 8861961 PMC452158

[46] Chagne D , Carlisle CM, Blond C et al. Mapping a candidate gene (MdMYB10) for red flesh and foliage colour in apple. *BMC Genomics.* 2007;8:212. 17608951 10.1186/1471-2164-8-212 PMC1939713

[47] Chagne D , Wang KL, Espley RV et al. An ancient duplication of apple MYB transcription factors is responsible for novel red fruit-flesh phenotypes. *Plant Physiol.* 2013;161:225–39. 23096157 10.1104/pp.112.206771 PMC3532254

[48] Espley RV , Brendolise C, Chagné D et al. Multiple repeats of a promoter segment causes transcription factor autoregulation in red apples. *Plant Cell.* 2009;21:168–83. 19151225 10.1105/tpc.108.059329 PMC2648084

[49] Espley RV , Hellens RP, Putterill J et al. Red colouration in apple fruit is due to the activity of the MYB transcription factor, MdMYB10. *Plant J.* 2007;49:414–27. 17181777 10.1111/j.1365-313X.2006.02964.x PMC1865000

[50] An JP , Zhang XW, Bi SQ et al. The ERF transcription factor MdERF38 promotes drought stress-induced anthocyanin biosynthesis in apple. *Plant J.* 2020;101:573–89. 31571281 10.1111/tpj.14555

[51] Liu Y , Zhang XW, Liu X et al. Phytochrome interacting factor MdPIF7 modulates anthocyanin biosynthesis and hypocotyl growth in apple. *Plant Physiol.* 2022;188:2342–63. 34983053 10.1093/plphys/kiab605 PMC8968312

[52] Wang S , Li LX, Zhang Z et al. Ethylene precisely regulates anthocyanin synthesis in apple via a module comprising MdEIL1, MdMYB1, and MdMYB17. *Hortic Res.* 2022;9:uhac034. 35184186 10.1093/hr/uhac034 PMC9039505

[53] Wang S , Wang T, Li Q et al. Phosphorylation of MdERF17 by MdMPK4 promotes apple fruit Peel Degreening during light/dark transitions. *Plant Cell.* 2022;34:1980–2000. 35166845 10.1093/plcell/koac049 PMC9048921

[54] Ma H , Yang T, Li Y et al. The long noncoding RNA MdLNC499 bridges MdWRKY1 and MdERF109 function to regulate early-stage light-induced anthocyanin accumulation in apple fruit. *Plant Cell.* 2021;33:3309–30. 34270784 10.1093/plcell/koab188 PMC8505877

[55] Yu J , Qiu K, Sun W et al. A long non-coding RNA functions in high-light-induced anthocyanin accumulation in apple by activating ethylene synthesis. *Plant Physiol.* 2022;189:66–83. 35148400 10.1093/plphys/kiac049 PMC9070812

[56] Li C , Wu J, Hu KD et al. PyWRKY26 and PybHLH3 cotargeted the PyMYB114 promoter to regulate anthocyanin biosynthesis and transport in red-skinned pears. *Hortic Res.* 2020;7:37. 32194973 10.1038/s41438-020-0254-z PMC7072072

[57] Medina-Puche L , Cumplido-Laso G, Amil-Ruiz F et al. MYB10 plays a major role in the regulation of flavonoid/phenylpropanoid metabolism during ripening of Fragaria x ananassa fruits. *J Exp Bot.* 2014;65:401–17. 24277278 10.1093/jxb/ert377

[58] Lin-Wang K , McGhie TK, Wang M et al. Engineering the anthocyanin regulatory complex of strawberry (Fragaria vesca). *Front Plant Sci.* 2014;5:651. 25477896 10.3389/fpls.2014.00651 PMC4237049

[59] Fei X , Ma Y, Hu H et al. Transcriptome analysis and GC-MS profiling of key genes in fatty acid synthesis of Zanthoxylum bungeanum seeds. *Ind Crop Prod.* 2020;156:112870.

[60] Li H , Li Y, Yu J et al. MdMYB8 is associated with flavonol biosynthesis via the activation of the MdFLS promoter in the fruits of Malus crabapple. *Hortic Res.* 2020;7:19. 32025322 10.1038/s41438-020-0238-z PMC6994661

[61] Zhang AD , Wang WQ, Tong Y et al. Transcriptome analysis identifies a zinc finger protein regulating starch degradation in kiwifruit. *Plant Physiol.* 2018;178:850–63. 30135096 10.1104/pp.18.00427 PMC6181057

[62] Wang R , Shu P, Zhang C et al. Integrative analyses of metabolome and genome-wide transcriptome reveal the regulatory network governing flavor formation in kiwifruit (Actinidia chinensis). *New Phytol.* 2022;233:373–89. 34255862 10.1111/nph.17618

[63] Li S , Zachgo S. TCP3 interacts with R2R3-MYB proteins, promotes flavonoid biosynthesis and negatively regulates the auxin response in Arabidopsis thaliana. *Plant J.* 2013;76:901–13. 24118612 10.1111/tpj.12348

[64] Dubos C , le Gourrierec J, Baudry A et al. MYBL2 is a new regulator of flavonoid biosynthesis in Arabidopsis thaliana. *Plant J.* 2008;55:940–53. 18532978 10.1111/j.1365-313X.2008.03564.x

[65] Zhou H , Lin-Wang K, Wang F et al. Activator-type R2R3-MYB genes induce a repressor-type R2R3-MYB gene to balance anthocyanin and proanthocyanidin accumulation. *New Phytol.* 2019;221:1919–34. 30222199 10.1111/nph.15486

[66] Cavallini E , Matus JT, Finezzo L et al. The Phenylpropanoid pathway is controlled at different branches by a set of R2R3-MYB C2 repressors in grapevine. *Plant Physiol.* 2015;167:1448–70. 25659381 10.1104/pp.114.256172 PMC4378173

[67] Lin-Wang K , Micheletti D, Palmer J et al. High temperature reduces apple fruit colour via modulation of the anthocyanin regulatory complex. *Plant Cell Environ.* 2011;34:1176–90. 21410713 10.1111/j.1365-3040.2011.02316.x

[68] Parenicova L , de Folter S, Kieffer M et al. Molecular and phylogenetic analyses of the complete MADS-box transcription factor family in Arabidopsis: new openings to the MADS world. *Plant Cell.* 2003;15:1538–51. 12837945 10.1105/tpc.011544 PMC165399

[69] Wang N , Liu W, Zhang T et al. Transcriptomic analysis of red-fleshed apples reveals the novel role of MdWRKY11 in flavonoid and anthocyanin biosynthesis. *J Agric Food Chem.* 2018;66:7076–86. 29909630 10.1021/acs.jafc.8b01273

[70] Benjamini Y , Hochberg Y. Controlling the false discovery rate - a practical and powerful approach to multiple testing. *J R Stat Soc Series B.* 1995;57:289–300.

[71] Anders S , Huber W. Differential expression analysis for sequence count data. *Genome Biol.* 2010;11:R106. 20979621 10.1186/gb-2010-11-10-r106 PMC3218662

[72] Livak KJ , Schmittgen TD. Analysis of relative gene expression data using real-time quantitative PCR and the 2(T)(-Delta Delta C) method. *Methods.* 2001;25:402–8. 11846609 10.1006/meth.2001.1262

[73] Tamura K , Peterson D, Peterson N et al. MEGA5: molecular evolutionary genetics analysis using maximum likelihood, evolutionary distance, and maximum parsimony methods. *Mol Biol Evol.* 2011;28:2731–9. 21546353 10.1093/molbev/msr121 PMC3203626

[74] Hellens RP , Allan AC, Friel EN et al. Transient expression vectors for functional genomics, quantification of promoter activity and RNA silencing in plants. *Plant Methods.* 2005;1:13. 16359558 10.1186/1746-4811-1-13 PMC1334188

[75] Chen HM , Zou Y, Shang Y et al. Firefly luciferase complementation imaging assay for protein-protein interactions in plants. *Plant Physiol.* 2008;146:368–76. 18065554 10.1104/pp.107.111740 PMC2245818

