# Supplementary material for: Interaction of AcMADS68 with transcription factors regulates anthocyanin biosynthesis in red-fleshed kiwifruit
Source: Hortic Res. 2022 Nov 15;10(2):uhac252. doi: 10.1093/hr/uhac252 (PMC9896601; doi:10.1093/hr/uhac252)
Supplement: Web_Material_uhac252 [file web_material_uhac252.zip › Figure S1-6.pdf]

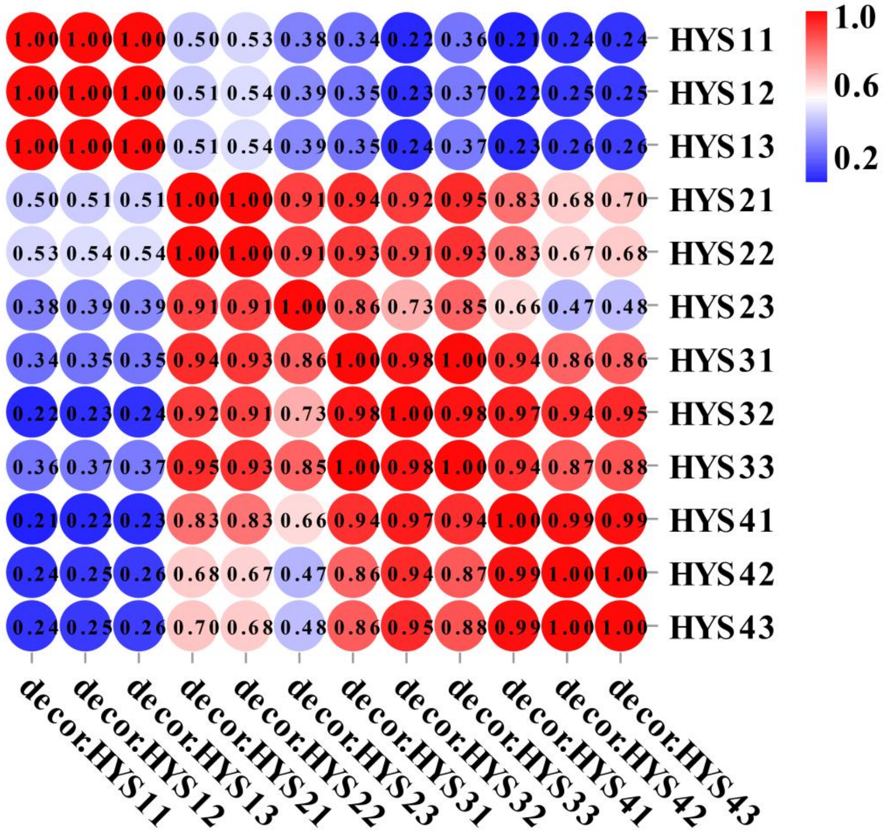

Figure S1 Heat map showing the correlations between biological replicates. The PCC (Pearson correlation coefficient) values are quantitative indicators of relative expression levels of all genes in each sample.

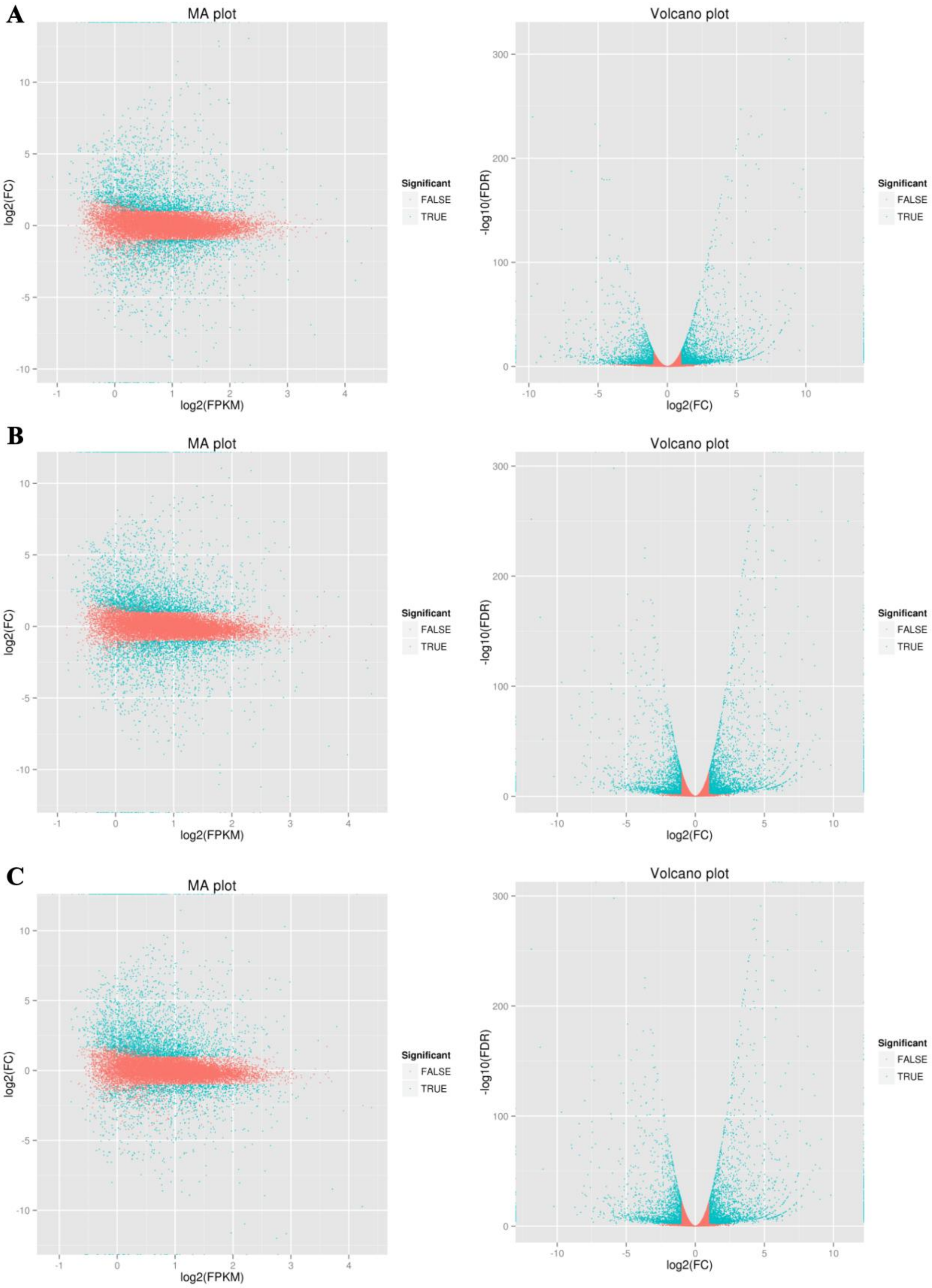

Figure S2 MA plot and volcano plot visualizing the DEGs between different stages. (A) S1 vs. S2, (B) S1 vs. S3, and (C) S1 vs. S4.

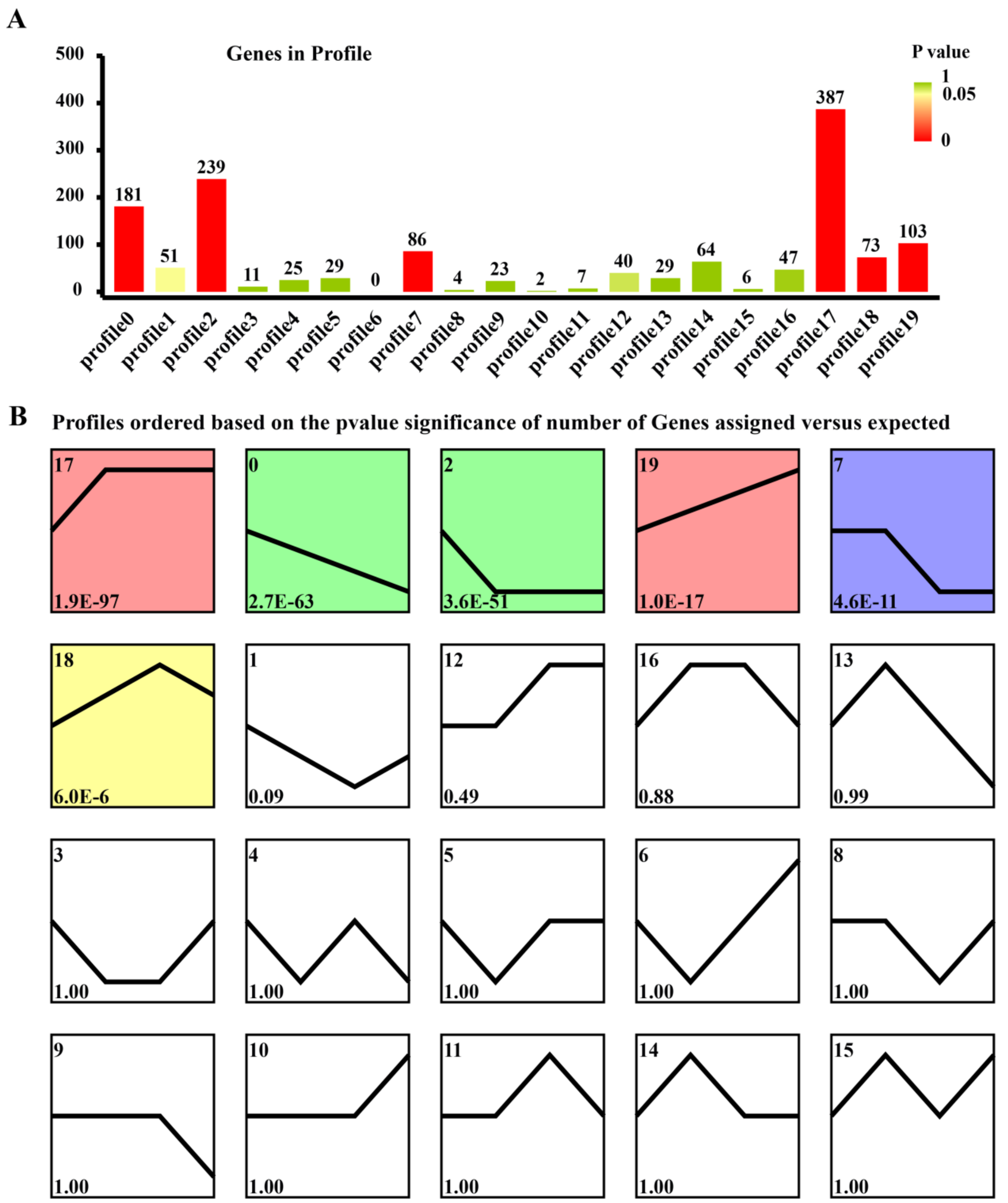

Figure S3 The results of trend analysis. (A) Gene number in each profile. (B) Profiles ordered based on the *p*-value significance of the number of genes assigned versus the number expected.

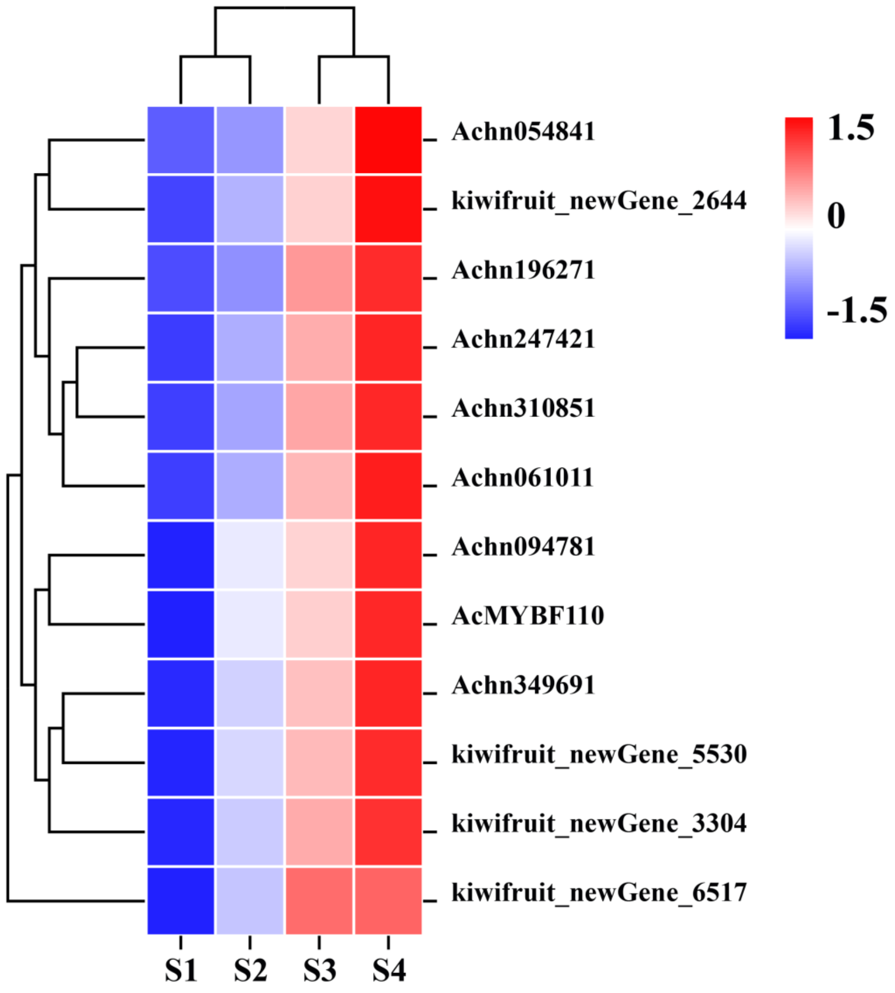

Figure S4 The FPKM values of 12 TFs from profile 19 in developing fruit.

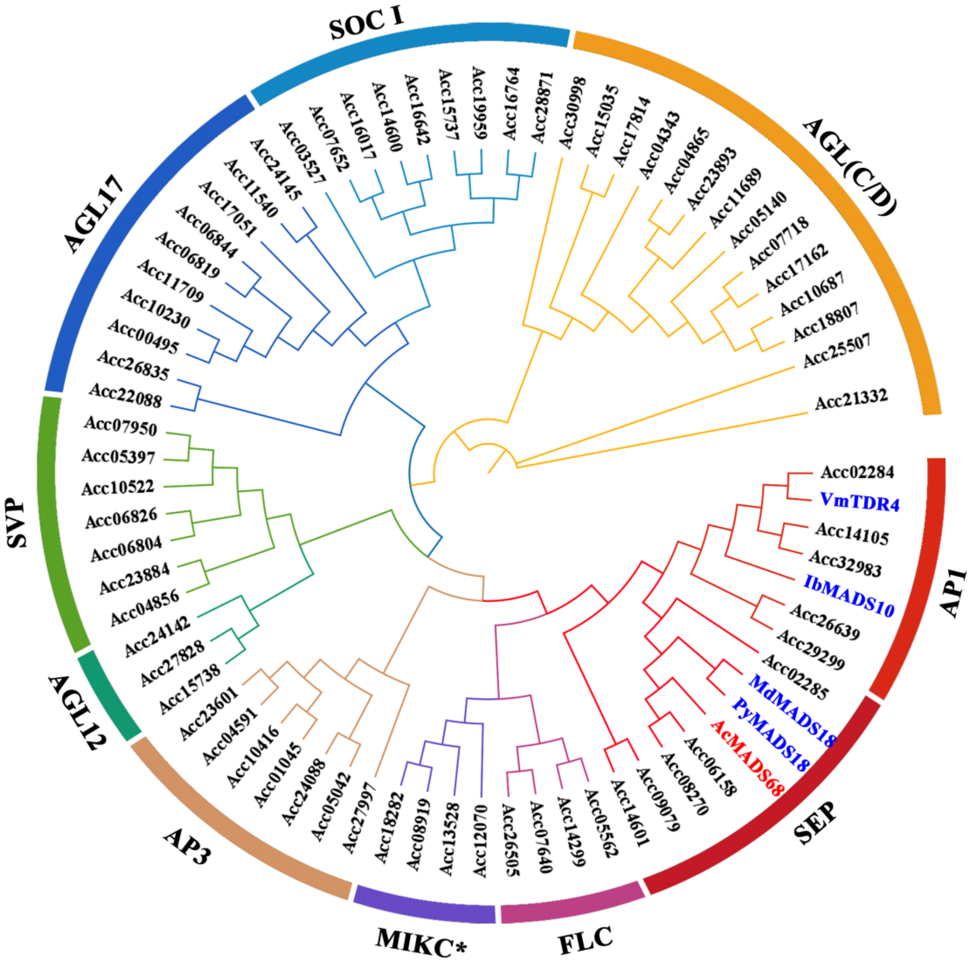

Figure S5 A phylogenetic tree of 69 kiwifruit MADS TFs and other published anthocyanin-related MADS TFs.

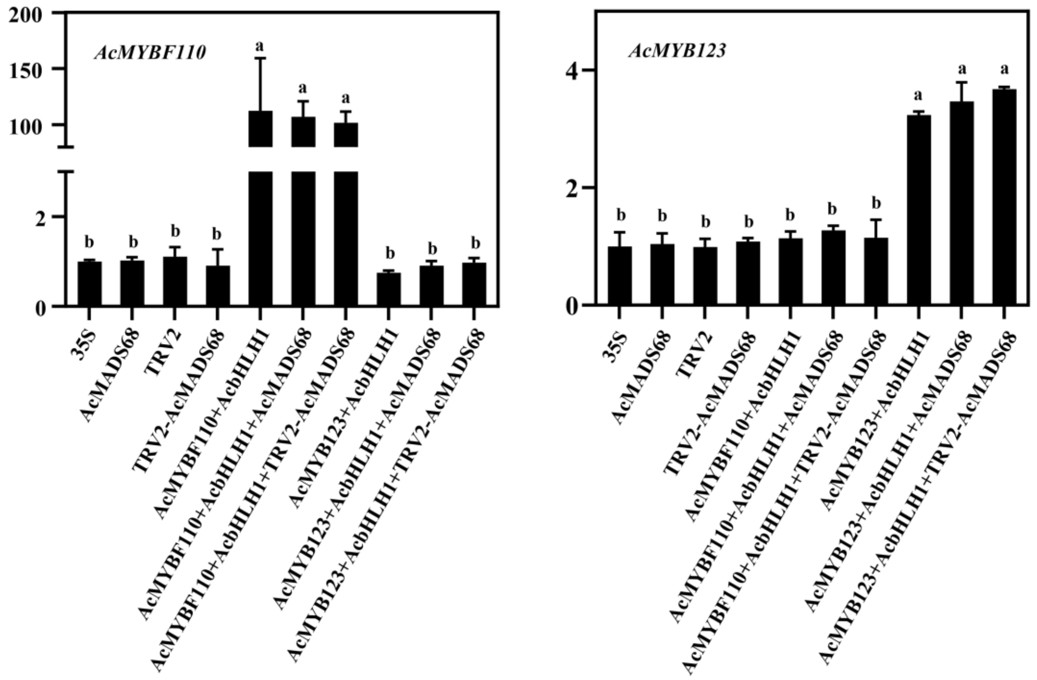

Figure S6 Expression levels of *AcMYBF110* and *AcMYB123* in tested *A. arguta* fruit.
